# Chlorido{2,2′-[propane-1,3-diylbis(nitrilo­methyl­idyne)]diphenolato-κ^4^ *O*,*N*,*N*′,*O*′}manganese(III)

**DOI:** 10.1107/S1600536809005352

**Published:** 2009-02-21

**Authors:** Ming-Jie Li, Peng-Fei Yan, Guang-Ming Li, Hong-Feng Li

**Affiliations:** a School of Chemistry and Materials Science, Heilongjiang University, Harbin 150080, People’s Republic of China

## Abstract

In the title complex, [Mn(C_17_H_16_N_2_O_2_)Cl], the Mn^III^ ion is coordinated by two O [Mn—O = 1.719 (2) and 1.813 (2) Å] and two N [Mn—N = 1.824 (2) and 1.931 (2) Å] atoms from the tetra­dentate Schiff base ligand and a chloride anion [Mn—Cl = 2.9634 (16) Å] in a square-pyramidal geometry. In the ligand, the two benzene rings form a dihedral angle of 68.06 (5)°.

## Related literature

For a similar manganese complex of the same Schiff base, see: Watkinson *et al.* (1999 ▶).
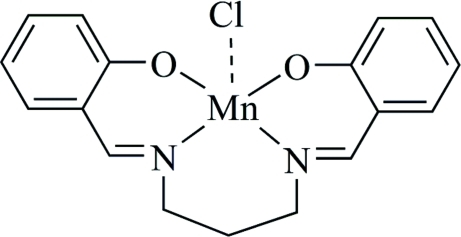


## Experimental

### 

#### Crystal data

[Mn(C_17_H_16_N_2_O_2_)Cl]
*M* *_r_* = 370.71
Orthorhombic, 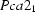

*a* = 10.428 (3) Å
*b* = 12.067 (4) Å
*c* = 12.530 (5) Å
*V* = 1576.6 (10) Å^3^
*Z* = 4
Mo *K*α radiation
μ = 1.02 mm^−1^
*T* = 291 K
0.19 × 0.17 × 0.12 mm

#### Data collection

Rigaku R-AXIS RAPID diffractometer
Absorption correction: multi-scan (*ABSCOR*; Higashi, 1995 ▶) *T*_min_ = 0.830, *T*_max_ = 0.889
11321 measured reflections
2689 independent reflections
2526 reflections with *I* > 2σ(*I*)
*R*_int_ = 0.032

#### Refinement

*R*[*F* ^2^ > 2σ(*F* ^2^)] = 0.035
*wR*(*F* ^2^) = 0.094
*S* = 1.05
2689 reflections
208 parameters
1 restraint
H-atom parameters constrained
Δρ_max_ = 0.44 e Å^−3^
Δρ_min_ = −0.31 e Å^−3^
Absolute structure: Flack (1983 ▶), 1227 Friedel pairs
Flack parameter: −0.01 (2)

### 

Data collection: *RAPID-AUTO* (Rigaku, 1998 ▶); cell refinement: *RAPID-AUTO*; data reduction: *CrystalClear* (Rigaku/MSC, 2002 ▶); program(s) used to solve structure: *SHELXS97* (Sheldrick, 2008 ▶); program(s) used to refine structure: *SHELXL97* (Sheldrick, 2008 ▶); molecular graphics: *SHELXTL* (Sheldrick, 2008 ▶); software used to prepare material for publication: *SHELXL97*.

## Supplementary Material

Crystal structure: contains datablocks I, global. DOI: 10.1107/S1600536809005352/cv2500sup1.cif

Structure factors: contains datablocks I. DOI: 10.1107/S1600536809005352/cv2500Isup2.hkl

Additional supplementary materials:  crystallographic information; 3D view; checkCIF report

## Figures and Tables

**Fig. 1.**
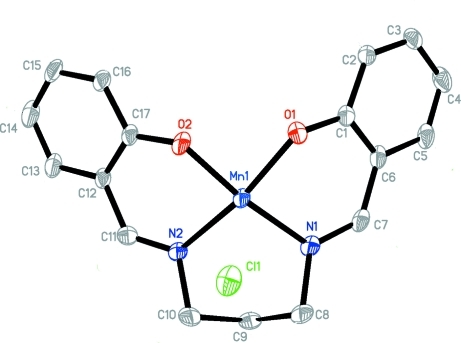
The molecular structure of (I), showing 30% probability displacement ellipsoids.

## supplementary crystallographic information

### Comment

In the title compound (Fig. 1), the tetradentate Schiff base ligand links Mn atom into a mononuclear complex through two phenolate O atoms and two N atoms with the bond lengths similar to those reported for another manganese complex of the same ligand (Watkinson *et al.*, 1999). The Mn^III^ center is five-coordinate by two nitrogen atoms and two oxygen atoms from the ligand and one chlorine anion in a square-pyramidal geometry.

### Experimental

The title complex was obtained by the treatment of manganese(III) chloride tetrahydrate with the Schiff base in methanol. The first two reactants were refluxed for 1 h. The reaction mixture was cooled and filtered; Diethyl ether was allowed to diffuse slowly into the solution of the filtrate. Single crystals were obtained after several days. Analysis: calculated for C_17_H_16_MnN_2_O_2_Cl: C, 55.08; H, 4.35; Mn, 14.82; N, 7.56; found: C, 54.98; H, 4.39; N, 7.45; Mn, 14.28%.

### Refinement

H atoms bound to C atoms were placed in calculated positions and treated as riding on their parent atoms, with C—H = 0.93 Å (aromatic C), C—H = 0.97 Å (methylene C),and with *U*_iso_(H) = 1.2Ueq(C).

### Crystal data

**Table:**

[Mn(C_17_H_16_N_2_O_2_)Cl]	*F*(000) = 760
*M**_r_* = 370.71	*D*_x_ = 1.562 Mg m^−^^3^
Orthorhombic, *P**c**a*2_1_	Mo *K*α radiation, λ = 0.71073 Å
Hall symbol: P 2c -2ac	Cell parameters from 12324 reflections
*a* = 10.428 (3) Å	θ = 3.1–27.5°
*b* = 12.067 (4) Å	µ = 1.02 mm^−^^1^
*c* = 12.530 (5) Å	*T* = 291 K
*V* = 1576.6 (10) Å^3^	Block, black
*Z* = 4	0.19 × 0.17 × 0.12 mm

### Data collection

**Table:**

Rigaku R-AXIS RAPID diffractometer	2689 independent reflections
Radiation source: fine-focus sealed tube	2526 reflections with *I* > 2σ(*I*)
graphite	*R*_int_ = 0.032
ω scans	θ_max_ = 25.0°, θ_min_ = 3.1°
Absorption correction: multi-scan (*ABSCOR*; Higashi, 1995)	*h* = −12→12
*T*_min_ = 0.830, *T*_max_ = 0.889	*k* = −14→14
11321 measured reflections	*l* = −13→13

### Refinement

**Table:**

Refinement on *F*^2^	Secondary atom site location: difference Fourier map
Least-squares matrix: full	Hydrogen site location: inferred from neighbouring sites
*R*[*F*^2^ > 2σ(*F*^2^)] = 0.035	H-atom parameters constrained
*wR*(*F*^2^) = 0.094	*w* = 1/[σ^2^(*F*_o_^2^) + (0.0666*P*)^2^ + 0.1785*P*] where *P* = (*F*_o_^2^ + 2*F*_c_^2^)/3
*S* = 1.05	(Δ/σ)_max_ = 0.001
2689 reflections	Δρ_max_ = 0.44 e Å^−^^3^
208 parameters	Δρ_min_ = −0.30 e Å^−^^3^
1 restraint	Absolute structure: Flack (1983), 1227 Friedel pairs
Primary atom site location: structure-invariant direct methods	Flack parameter: −0.01 (2)

### Special details

**Table:**

Geometry. All e.s.d.'s (except the e.s.d. in the dihedral angle between two l.s. planes) are estimated using the full covariance matrix. The cell e.s.d.'s are taken into account individually in the estimation of e.s.d.'s in distances, angles and torsion angles; correlations between e.s.d.'s in cell parameters are only used when they are defined by crystal symmetry. An approximate (isotropic) treatment of cell e.s.d.'s is used for estimating e.s.d.'s involving l.s. planes.
Refinement. Refinement of *F*^2^ against ALL reflections. The weighted *R*-factor *wR* and goodness of fit *S* are based on *F*^2^, conventional *R*-factors *R* are based on *F*, with *F* set to zero for negative *F*^2^. The threshold expression of *F*^2^ > σ(*F*^2^) is used only for calculating *R*-factors(gt) *etc*. and is not relevant to the choice of reflections for refinement. *R*-factors based on *F*^2^ are statistically about twice as large as those based on *F*, and *R*- factors based on ALL data will be even larger.

### Fractional atomic coordinates and isotropic or equivalent isotropic displacement parameters (Å^2^)

**Table:**

	*x*	*y*	*z*	*U*_iso_*/*U*_eq_	
C1	0.8363 (3)	0.4885 (3)	0.7136 (3)	0.0330 (8)	
C2	0.9063 (3)	0.4297 (3)	0.6323 (3)	0.0383 (8)	
H1	0.9918	0.4508	0.6257	0.046*	
C3	0.8679 (3)	0.3466 (3)	0.5617 (3)	0.0434 (9)	
H2	0.9238	0.3167	0.5115	0.052*	
C4	0.7560 (5)	0.3148 (3)	0.5694 (5)	0.0550 (12)	
H3	0.7204	0.2607	0.5256	0.066*	
C5	0.6881 (3)	0.3678 (3)	0.6502 (4)	0.0506 (11)	
H4	0.6045	0.3426	0.6589	0.061*	
C6	0.7247 (3)	0.4555 (3)	0.7232 (3)	0.0365 (9)	
C7	0.6476 (3)	0.5107 (3)	0.8041 (3)	0.0365 (8)	
H5	0.5738	0.4717	0.8224	0.044*	
C8	0.5664 (3)	0.6436 (3)	0.9321 (3)	0.0378 (8)	
H7	0.6039	0.6538	1.0022	0.045*	
H6	0.4934	0.5941	0.9376	0.045*	
C9	0.5303 (3)	0.7500 (3)	0.8835 (3)	0.0393 (8)	
H8	0.5342	0.7425	0.8065	0.047*	
H9	0.4421	0.7665	0.9024	0.047*	
C10	0.6086 (3)	0.8410 (3)	0.9145 (3)	0.0397 (9)	
H10	0.5632	0.9106	0.9056	0.048*	
H11	0.6352	0.8340	0.9884	0.048*	
C11	0.7546 (3)	0.9233 (3)	0.8068 (3)	0.0341 (8)	
H12	0.7055	0.9853	0.8231	0.041*	
C12	0.8596 (3)	0.9429 (3)	0.7439 (3)	0.0342 (8)	
C13	0.8794 (3)	1.0423 (3)	0.6866 (4)	0.0468 (10)	
H13	0.8180	1.0976	0.6947	0.056*	
C14	0.9749 (4)	1.0638 (3)	0.6237 (4)	0.0499 (10)	
H14	0.9815	1.1286	0.5839	0.060*	
C15	1.0592 (3)	0.9870 (3)	0.6221 (3)	0.0455 (10)	
H15	1.1323	0.9960	0.5805	0.055*	
C16	1.0440 (3)	0.8901 (3)	0.6812 (3)	0.0422 (9)	
H16	1.1107	0.8390	0.6787	0.051*	
C17	0.9428 (3)	0.8637 (3)	0.7408 (3)	0.0320 (8)	
Cl1	0.82072 (8)	0.70229 (8)	1.06882 (7)	0.0435 (2)	
Mn1	0.79832 (4)	0.69247 (3)	0.83324 (5)	0.03055 (16)	
N1	0.6603 (2)	0.6027 (2)	0.8558 (2)	0.0319 (7)	
N2	0.7160 (2)	0.8353 (2)	0.8449 (3)	0.0303 (6)	
O1	0.87583 (19)	0.56992 (19)	0.7799 (2)	0.0435 (6)	
O2	0.92804 (19)	0.76783 (19)	0.7896 (2)	0.0457 (7)	

### Atomic displacement parameters (Å^2^)

**Table:**

	*U*^11^	*U*^22^	*U*^33^	*U*^12^	*U*^13^	*U*^23^
C1	0.0170 (15)	0.0230 (17)	0.059 (2)	0.0016 (12)	−0.0073 (14)	0.0037 (14)
C2	0.0165 (15)	0.0260 (17)	0.072 (3)	0.0026 (13)	−0.0026 (16)	0.0053 (15)
C3	0.0275 (17)	0.0269 (17)	0.076 (3)	0.0054 (14)	−0.0028 (18)	−0.0031 (17)
C4	0.0323 (18)	0.0296 (17)	0.103 (4)	−0.0024 (16)	−0.012 (2)	−0.015 (2)
C5	0.0223 (17)	0.0280 (18)	0.101 (3)	−0.0069 (14)	−0.0072 (19)	0.004 (2)
C6	0.0206 (17)	0.0237 (16)	0.065 (3)	−0.0017 (13)	−0.0096 (15)	0.0087 (15)
C7	0.0142 (13)	0.0296 (17)	0.066 (2)	−0.0035 (12)	−0.0056 (14)	0.0123 (16)
C8	0.0157 (14)	0.044 (2)	0.053 (2)	−0.0016 (13)	0.0046 (14)	0.0005 (16)
C9	0.0135 (14)	0.044 (2)	0.060 (2)	0.0056 (13)	0.0084 (15)	−0.0050 (16)
C10	0.0215 (15)	0.0381 (19)	0.060 (2)	0.0082 (14)	0.0048 (15)	−0.0085 (16)
C11	0.0183 (13)	0.0324 (17)	0.052 (2)	0.0076 (13)	−0.0021 (14)	−0.0019 (14)
C12	0.0198 (15)	0.0224 (15)	0.060 (2)	−0.0009 (12)	−0.0024 (15)	−0.0026 (15)
C13	0.0268 (18)	0.0245 (18)	0.089 (3)	−0.0020 (13)	−0.005 (2)	0.0033 (18)
C14	0.035 (2)	0.0296 (18)	0.085 (3)	−0.0068 (16)	−0.013 (2)	0.0107 (18)
C15	0.0271 (17)	0.040 (2)	0.069 (3)	−0.0159 (16)	−0.0009 (17)	0.0012 (18)
C16	0.0193 (16)	0.0310 (18)	0.076 (3)	−0.0047 (13)	−0.0011 (17)	−0.0065 (18)
C17	0.0171 (15)	0.0253 (17)	0.054 (2)	−0.0036 (12)	−0.0080 (14)	−0.0018 (15)
Cl1	0.0340 (4)	0.0450 (5)	0.0516 (6)	−0.0052 (4)	−0.0144 (4)	0.0071 (4)
Mn1	0.0114 (2)	0.0231 (2)	0.0571 (3)	0.00044 (15)	−0.0008 (2)	0.0015 (3)
N1	0.0106 (10)	0.0306 (14)	0.0544 (19)	0.0022 (9)	−0.0040 (11)	0.0068 (13)
N2	0.0120 (10)	0.0291 (13)	0.0499 (17)	0.0028 (9)	−0.0012 (12)	−0.0002 (15)
O1	0.0079 (9)	0.0278 (12)	0.0949 (18)	0.0026 (8)	−0.0058 (11)	−0.0085 (12)
O2	0.0087 (9)	0.0268 (12)	0.101 (2)	0.0014 (8)	−0.0015 (11)	0.0066 (13)

### Geometric parameters (Å, °)

**Table:**

C1—C6	1.236 (5)	C10—H10	0.9700
C1—O1	1.351 (4)	C10—H11	0.9700
C1—C2	1.439 (5)	C11—N2	1.232 (4)
C2—C3	1.397 (5)	C11—C12	1.370 (5)
C2—H1	0.9300	C11—H12	0.9300
C3—C4	1.232 (6)	C12—C17	1.292 (5)
C3—H2	0.9300	C12—C13	1.413 (5)
C4—C5	1.391 (7)	C13—C14	1.296 (6)
C4—H3	0.9300	C13—H13	0.9300
C5—C6	1.449 (6)	C14—C15	1.277 (5)
C5—H4	0.9300	C14—H14	0.9300
C6—C7	1.456 (5)	C15—C16	1.394 (5)
C7—N1	1.291 (4)	C15—H15	0.9300
C7—H5	0.9300	C16—C17	1.331 (5)
C8—N1	1.455 (4)	C16—H16	0.9300
C8—C9	1.470 (5)	C17—O2	1.317 (4)
C8—H7	0.9700	Cl1—Mn1	2.9634 (16)
C8—H6	0.9700	Mn1—O2	1.719 (2)
C9—C10	1.423 (5)	Mn1—O1	1.813 (2)
C9—H8	0.9700	Mn1—N1	1.824 (2)
C9—H9	0.9700	Mn1—N2	1.931 (2)
C10—N2	1.421 (4)		
			
C6—C1—O1	117.5 (3)	N2—C11—C12	129.3 (3)
C6—C1—C2	112.9 (3)	N2—C11—H12	115.4
O1—C1—C2	129.6 (3)	C12—C11—H12	115.4
C3—C2—C1	131.0 (3)	C17—C12—C11	115.2 (3)
C3—C2—H1	114.5	C17—C12—C13	121.0 (3)
C1—C2—H1	114.5	C11—C12—C13	123.8 (3)
C4—C3—C2	116.5 (4)	C14—C13—C12	126.3 (4)
C4—C3—H2	121.8	C14—C13—H13	116.9
C2—C3—H2	121.8	C12—C13—H13	116.9
C3—C4—C5	113.3 (4)	C15—C14—C13	113.1 (4)
C3—C4—H3	123.3	C15—C14—H14	123.4
C5—C4—H3	123.3	C13—C14—H14	123.4
C4—C5—C6	131.4 (3)	C14—C15—C16	121.5 (4)
C4—C5—H4	114.3	C14—C15—H15	119.3
C6—C5—H4	114.3	C16—C15—H15	119.3
C1—C6—C5	114.9 (4)	C17—C16—C15	126.1 (3)
C1—C6—C7	116.1 (3)	C17—C16—H16	116.9
C5—C6—C7	128.9 (3)	C15—C16—H16	116.9
N1—C7—C6	133.3 (3)	C12—C17—O2	123.9 (3)
N1—C7—H5	113.4	C12—C17—C16	111.8 (3)
C6—C7—H5	113.4	O2—C17—C16	124.3 (3)
N1—C8—C9	101.3 (3)	O2—Mn1—O1	87.90 (11)
N1—C8—H7	111.5	O2—Mn1—N1	169.92 (14)
C9—C8—H7	111.5	O1—Mn1—N1	85.65 (11)
N1—C8—H6	111.5	O2—Mn1—N2	84.35 (11)
C9—C8—H6	111.5	O1—Mn1—N2	162.58 (14)
H7—C8—H6	109.3	N1—Mn1—N2	99.68 (11)
C10—C9—C8	114.5 (3)	O2—Mn1—Cl1	103.51 (11)
C10—C9—H8	108.6	O1—Mn1—Cl1	111.39 (10)
C8—C9—H8	108.6	N1—Mn1—Cl1	86.08 (9)
C10—C9—H9	108.6	N2—Mn1—Cl1	85.64 (10)
C8—C9—H9	108.6	C7—N1—C8	123.5 (3)
H8—C9—H9	107.6	C7—N1—Mn1	120.9 (2)
N2—C10—C9	104.3 (3)	C8—N1—Mn1	115.6 (2)
N2—C10—H10	110.9	C11—N2—C10	117.0 (3)
C9—C10—H10	110.9	C11—N2—Mn1	126.5 (2)
N2—C10—H11	110.9	C10—N2—Mn1	116.1 (2)
C9—C10—H11	110.9	C1—O1—Mn1	133.14 (19)
H10—C10—H11	108.9	C17—O2—Mn1	134.8 (2)
